# Creep characteristics and damage model of coal–rock combinations with different height ratios

**DOI:** 10.1038/s41598-023-49841-4

**Published:** 2023-12-27

**Authors:** Junguang Wang, Song Yang, Yanming Qi, Yiran Cong

**Affiliations:** 1 School of Mechanics and Engineering, Liaoning Technical University, Fuxin, 123000 People’s Republic of China; 2 Liaoning Province Nonferrous Geology 101 Team Co. Ltd, Fushun, 113006 People’s Republic of China; 3 Qingdao Feiyang Human Resources Development Co. Ltd, Qingdao, 266041 People’s Republic of China

**Keywords:** Engineering, Materials science

## Abstract

Numerous coal pillars are left after the coal mining process. The composite structure comprising a roof and coal pillar has prominent creep characteristics, which threaten safe underground mining. Therefore, the creep characteristics of coal-rock combinations should be studied to ensure the safety of quarry and surface. Uniaxial creep tests under static load axial pressure and different height ratios were performed using a self-designed rock creep disturbance test device to determine the effect of height ratio and axial pressure on the creep characteristics of coal–rock combinations. From the test results, a creep damage model for coal–rock combinations was established by combining the elastomer, fractional Kelvin body, plastic body, Abel dashpot, and modified nonlinear viscoplastic body; introducing damage variables D related to stress, height ratio, and time; and deriving a one-dimensional creep equation. An improved nonlinear least squares method based on pattern search was utilized to invert the creep parameters. The results of the creep equation calculation were fitted with the experimental results with good results. The creep curve with a height ratio of 2:1 was predicted with good results. The research results provide theoretical references for long-term stability analysis of rock engineering.

## Introduction

**Figure 1:**
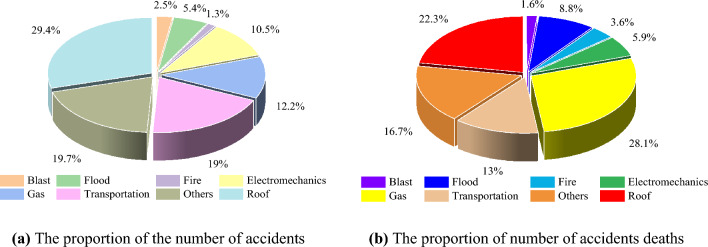
HYPERLINK "sps:id::fig1||locator::gr1||MediaObject::0"The proportion of total mine accidents and fatalities in each category^2^.

Coal resources remain an important basic energy source for industrial development, economic stability, and social progress in China^1^. With the rapid mining of coal resources, mine accidents occur frequently. Figure 1 shows the proportion of total mine accidents and fatalities in each category^2,3^. As can be seen from Fig. 1, roof accidents account for the highest percentage and the number of fatalities is second only to gas accidents.

Setting coal pillars is a primary method for preventing disasters and ensuring the safe and efficient mining of coal resources, as well as the safety of surface buildings and personnel^4^. The stability of the coal-rock combination structures, which involve coal pillars and roof rocks, is crucial for safe mining in underground coal mines, as well as for the safety of surface personnel and buildings. In the short term, the composite structure can maintain stability. However, over time and under the long-term effect of overburden loading, it may deform due to its significant rheological properties, leading to damage instability and potentially serious disasters^5,6^. Creep, which is one of the main manifestations of rheology^7–9^, can have a significant impact on the stability of the coal-rock combination structures. The results of creep studies on individual coal-rock combinations do not fully reflect the actual working conditions. Therefore, it is important to study the creep characteristics of coal-rock combinations to ensure the safety of quarry and surface operations.

Scholars from various countries have extensively researched coal-rock combinations. In 1979, Petukhov and Linkov^10^ analyzed the stability of the postpeak section of the "roof-bottom-body" system. Xie^11^ developed a two-body mechanics model that considers the contact surface effect between engineering and geological bodies, providing new insights into studying composite structures. Reed Guy^12^ proposed including the height ratio in the system stability standard, which relates to the interaction between the coal pillar and the roof. Liu^13^ established two damage constitutive models for coal, connecting the damaged body with the Newton body in series, and uncovered the influence of rock on the mechanical behavior of coal in coal-rock combinations. Xue^14^ conducted a numerical simulation to compare the dynamic response of four coal-rock combination media under three different stress waves, observing variations in the reflected wave, incident wave, strain, and strain rate. Zuo^15^ investigated the deformation and damage behavior of rock-coal combinations with a soft coal interlayer using an MTS 815 testing machine. They discovered that the weak coal interlayer altered the overall damage pattern of the coal body in the combinations and reduced its overall stability.

Tan^16^ employed the particle flow code (PFC 2D) to perform numerical simulations of a uniaxial compression test on heterogeneous coal-rock combinations adhering to the Weibull distribution. Du^17^ executed both physical experiments and numerical simulations to study gas-charged coal-rock combinations under varied conditions, concentrating on composite dynamic disasters such as outburst-rockbursts and conducting a thorough analysis of their occurrence conditions and dynamic response features. Zhang^18^ carried out uniaxial compression and Brazilian splitting tests on coal-rock combinations and used the UDEC-Trigon method to create a corresponding numerical model, which helped determine the number, length, and macroscopic areas of cracks during fracture and destabilization processes, shedding light on the patterns of crack propagation and energy evolution. Wang^19^ introduced a strain-softening damage constitutive model solution method tailored for coal–rock combinations, capturing their stress–strain behavior with precision. Liu^20^ explored the impact of crack length and orientation on the mechanical characteristics and failure patterns of coal-rock combinations by conducting uniaxial compression tests on samples with prefabricated fissures.

Much of the current research on coal–rock combinations has been directed toward understanding rockburst phenomena, operating under the hypothesis that coal pillar instability during such events is an instantaneous process. Yet, it often transpires that coal pillar instability is heralded by rheological deformation, and the aggregate damage manifests as a time-delayed response rather than an abrupt occurrence. Hence, to assure lasting stability, it is vital to analyze the creep characteristics of the combined roof-coal pillar structure^5,21^. The height ratio is recognized as a crucial determinant of its deformation behavior. Chen^22^ elucidated the impact of height ratio on the mechanical properties and the resultant force effects in coal-rock combinations by performing uniaxial compression tests across varying height ratios. Also, Wang^23^ assessed the connection between height ratio and the triaxial failure attributes and mechanical parameters of coal-rock combinations, utilizing tests with varied height ratios. Ma^24^ leveraged PFC software to imitate uniaxial compression tests on coal–rock combinations with diverse height ratios, exploring the dynamics of crack evolution and energy fluctuations at different heights. Despite this knowledge base, scrutiny of the creep characteristics in coal–rock combinations has been scant in recent times. Consequently, addressing the gap in understanding the influence of creep on coal-rock combinations spanning different height ratios, and establishing an appropriate creep model tailored for these variations, stands out as an imperative scientific query warranting urgent investigation.

Creep exhibits significant temporal variability, and fractional calculus serves as a potent mathematical tool with an exceptional capability for retention. In recent times, the Abel dashpot, drawing from fractional order theory, has gained widespread recognition for its efficacy in analyzing creep models, culminating in a wealth of scholarly findings. The principal methodologies scholars have adopted to devise creep models can be compartmentalized as follows: (1) Researchers refine creep models by integrating viscous elements tailored by nonlinear functions^25^. (2) Models are expanded upon by serial and parallel incorporation of the traditional Abel dashpot^26,27^ or its adaptive variant, the variable-order Abel dashpot^28,29^, with pre-existing empirical constructions. (3) Damage variables are incorporated within the elemental blocks, either during the accelerated creep phase or pervasive throughout the entire creep phase, enabling the formulation of creep damage models^30,31^. (4) The combination of the above methods is also used^32–34^.

In summary, the exploration of creep properties and the development of a creep model specific to coal–rock combinations have not been thoroughly pursued, necessitating further investigation in the field. This study sets out with coal mine stability as the contextual framework, prepares samples of coal–rock combinations with varied height ratios, and employs an innovative rock creep testing apparatus to carry out uniaxial creep assays on these combinations under static axial load and different height ratios. A graduated loading technique is applied to elucidate the effect of height ratio and axial pressure on the coal–rock combinations' creep characteristics. Drawing on the empirical data and rock mechanics theories, the study introduces damage variables that intersect with stress, height ratio, and temporal factors to construct a fractional order creep damage model that characterizes the creep behavior of coal–rock combinations. The research aims to enhance understanding of the mechanical interrelations and destabilization mechanisms pertinent to coal-rock dynamics in the context of deep-mining operations.

## Creep test and result analysis of coal-rock combinations

### Testing equipment

**Figure 2:**
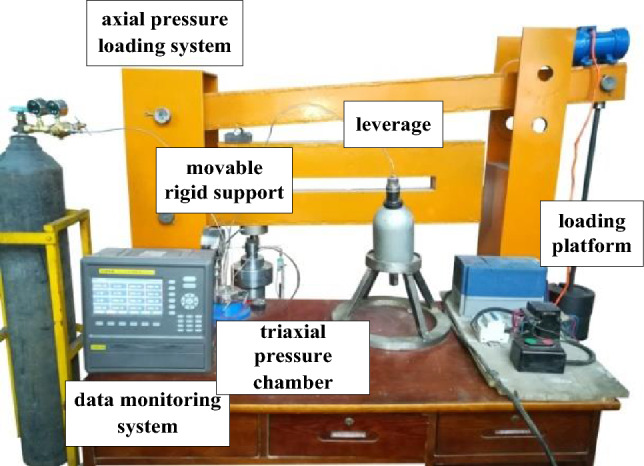
Schematic of the creep device.

A self-developed rock creep disturbance test device was used to perform the uniaxial creep test. This test device is equipped to operate uniaxial, triaxial, and applied disturbance creep tests. The creep test device consists of five parts: axial pressure loading system, confining pressure loading system, triaxial pressure chamber, disturbance loading system, and data monitoring system^8,9^, as shown in Fig. 2.

During the uniaxial creep test, the axial pressure loading system, the triaxial pressure chamber (this experiment involved the uniaxial creep test, so only the specimen is placed inside the pressure chamber without the need to apply confining pressure), and data monitoring system were utilized.

#### (1) Axial pressure loading system

The axial pressure loading system consists of three levers, a movable rigid support, and a loading platform. The principle of pressurization is accomplished by the secondary amplification action of the double lever, where weights are placed on the loading platform to increase the load. The middle of the lever is a movable rigid support that can be moved left and right, and the magnification is different for different locations. The magnification chosen for this experiment was calculated to be approximately 34.02 times.

#### (2) Data monitoring system

The data monitoring system uses a TOPRIE multichannel data logger. The logger can perform multichannel stress and displacement monitoring based on the sensor connection, recording once per second. It has an automatic internal storage function and can be connected to network devices for real-time monitoring, and the test data can be imported into the computer in table format for easy data processing.

### Specimen preparation

**Figure 3:**
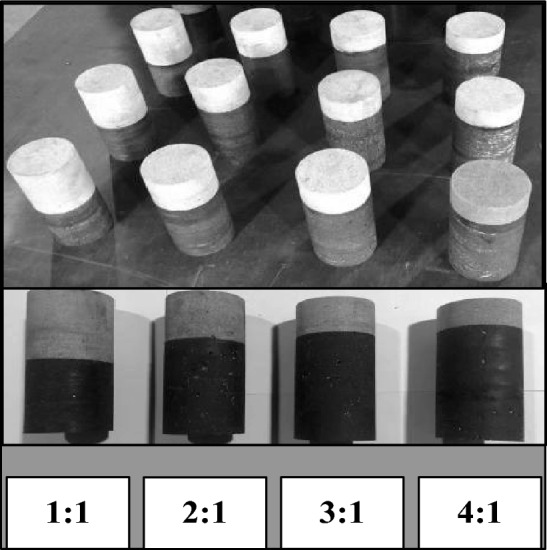
Specimens of coal-rock combinations.

Test specimens of coal and rock were procured from a coal mine located in Inner Mongolia, which underwent an initial sizing process before being drilled into cores using a precision coring apparatus. This produced coal and rock samples of various axial dimensions (20 mm, 25 mm, 33.3 mm, 50 mm, 66.7 mm, 75 mm, and 80 mm), each with a uniform diameter of 50 mm. To ensure planar uniformity and dimensional accuracy, the ends of the specimens were meticulously ground using a lapping machine, with the two terminal surfaces maintained in parallel alignment and a diameter discrepancy limited to 0.01 mm. Owing to its superior penetrating ability, exceptional adhesive properties, and resilience to high temperatures, mica adhesive was chosen as the binding agent for coupling coal and rock samples of assorted axial sizes, forming stratified assemblies with the rock atop and coal beneath. Standardized coal–rock combination specimens, measuring 50 mm in diameter and 100 mm in length, were then crafted to yield different height ratios of 1:1, 2:1, 3:1, and 4:1^35^, as shown in Fig. 3.

### Creep test process

**Figure 4:**
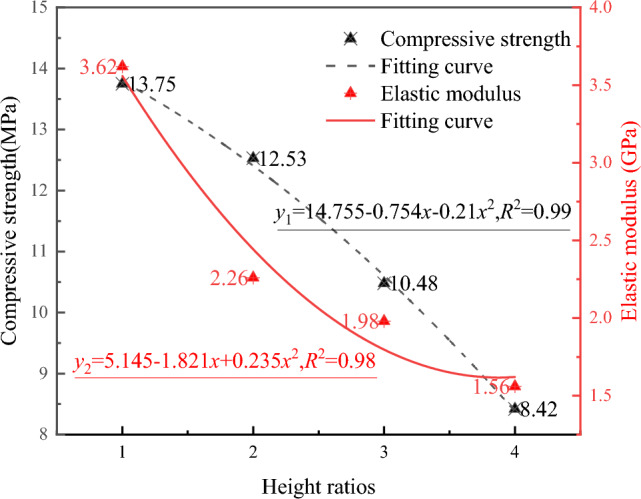
Graph of compressive strength and elastic modulus.

Before the creep tests, conventional uniaxial compression tests were performed on coal–rock combinations with different height ratios. The basic mechanical parameters under the test loading stress setting are obtained by calculating the basic mechanical parameters, as shown in Fig. 4, which shows that the compressive strength and elastic modulus decrease with the increase of height ratio. Because the increase in height ratio represents an increase in the overall proportion of the coal body, the overall stiffness of the composite coal rock body and compressive strength both decrease.

**Figure 5:**
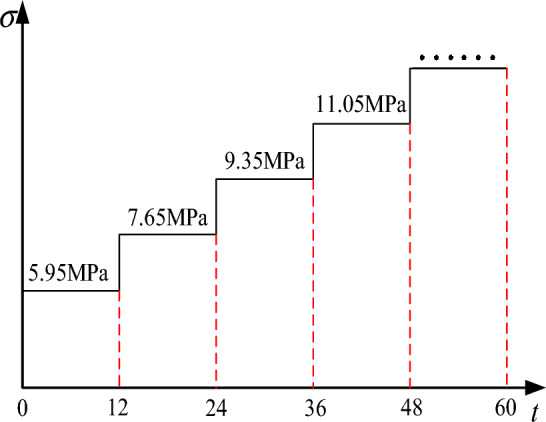
Loading scheme of creep test.

The creep test loading method for graded loading evaluates the effects of different axial stresses and height ratios through uniaxial creep tests. The creep test loading scheme was designed based on the uniaxial compression test results, as shown in Fig. 5. Starting at an initial stress of 6 MPa, reflecting the compressive strength depicted in Fig. 4, the loading sequence involved augmenting the load platform with 10 kg increments until the onset of specimen failure. Post-leverage enhancement, every addition was calculated to amplify the axial stress by 1.70 MPa.

The specific test procedure was as follows:

1. Place the test piece in the center of the pressure chamber, ensuring good contact between the top of the chamber and the lever, connect the sensor to the multi-channel data logger, and turn on the power.
2. Apply weights to the loading platform and observe the change in the stress value on the recorder until a stable value of 6 MPa is obtained, and then stop applying weights.
3. Record the loading time; maintain each load level for 12 h; and when the specimen is not unstable failure, apply a 10 kg weight until the specimen instability failure.
4. After the test, export the data of the multiplex data logger, and convert the recorded displacements into strains to facilitate a comparative analysis of creep data for different stress levels under specific height ratio conditions and creep data for different height ratios under specific stress level conditions. Remove the weights, take out the specimen, and prepare for the next set of tests.

### Creep test results analysis

**Figure 6:**
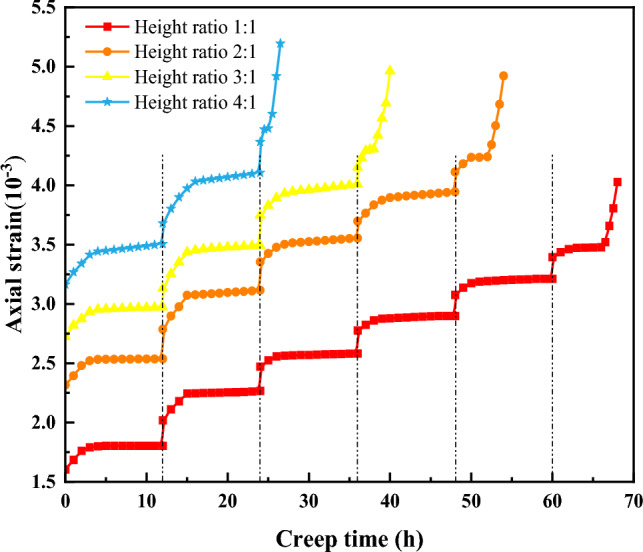
Creep test curves of coal–rock combinations with different height ratios.

The creep test curves of coal–rock combinations with different height ratios are shown in Fig. 6.

Figure 6 shows that the creep processes in coal-rock combinations with varying height ratios are similar. The specimen produces instantaneous deformation at the moment of loading and enters the initial creep stage under the action of low axial stress before tending to a steady state. When the applied axial stress exceeds the steady creep stress threshold, the initial creep stage occurs, followed by the steady creep stage after a period. For the specimens with height ratios of 1:1, 2:1, 3:1, and 4:1, the transition into the steady creep stage was observed at the 4th, 3rd, 2nd, and 1st stages of loading, respectively. When axial stresses passed the threshold for accelerated creep strain (viz., when the maximum load was applied), the sequence initiated with the initial creep stage, progressed to a steady stage quickly, and then to an accelerated stage, all preceding creep failure, which reflected the three stages of creep deformation. The specimens with height ratios of 1:1, 2:1, 3:1, and 4:1 were destroyed at level 6, level 5, level 4, and level 3 loads, respectively. As the height ratio increases, the ultimate load level that the coal–rock combinations can withstand decreases. With identical axial stress levels, the total deformation of the coal–rock combinations gradually increases with the increase in height ratio. Holding the same height ratio, total deformation gradually increased with ascending axial stress levels. Considering the entire creep trajectory, the aggregate duration of creep contracted with the rise in height ratio. Under the last stress level, the specimens exhibited all the three stages of creep (initial, steady, and accelerated creep stages) regardless of height ratio. With the increase in height ratio, the creep time also exhibited a decreasing trend: that is, the larger the proportion of the coal body part to the overall body of the combination, the faster the creep ended, which accelerated the destruction of the coal–rock combinations.

From the perspective of instantaneous deformation, under the same axial stress, the instantaneous deformation of the specimens with height ratios of 1:1, 2:1, 3:1, and 4:1 were 1.60 × 10^–3^, 2.31 × 10^–3^, 2.72 × 10^–3^, and 3.11 × 10^–3^. Under the first stage of loading, for example, the instantaneous deformation gradually increased with the height ratio. Under the 1st level load, the instantaneous deformation increased by 0.71 × 10^–3^ for the height ratio of 2:1 compared to that of the 1:1 height ratio, by 0.41 × 10^–3^ for the height ratio of 3:1 compared to that of the height ratio of 2:1, and by 0.39 × 10^–3^ for the height ratio of 4:1 compared to that of the height ratio of 3:1. This is attributable to the specimen with a height ratio of 2:1 being 1/6 larger than the coal body with a height ratio of 1:1, the specimen with a height ratio of 3:1 being 1/12 larger than the coal body with a height ratio of 2:1, and the specimen with a height ratio of 4:1 being 1/20 larger than the coal body with a height ratio of 3:1. The increasing trend in the coal body gradually decreased, and thus, the increase in the instantaneous deformation gradually decreased.

From the perspective of creep deformation, the creep deformation at six axial stress levels were 0.19 × 10^–3^, 0.24 × 10^–3^, 0.11 × 10^–3^, 0.12 × 10^–3^, 0.13 × 10^–3^, and 0.63 × 10^–3^ for a specimen with a height ratio of 1:1 for example. As the axial stress increased, the creep deformation tended to increase, decrease, and then increase because the coal–rock combinations are porous media with numerous internal pores. Moreover, after the first few levels of load application, the internal pores of the composite body are gradually compacted, and larger deformation occurs. The specimen is already compacted when the last few levels of load are applied, so less deformation occurs. At the last level of load application, the specimen cracks start to expand until the specimens are destroyed, and the deformation increases. Creep deformation responses of specimens with height ratios of 1:1, 2:1, 3:1, and 4:1 subjected to identical axial stress were recorded as 0.19 × 10^–3^, 0.22 × 10^–3^, 0.26 × 10^–3^, and 0.34 × 10^–3^ respectively at the initial loading stage, demonstrating a progressive increase correlating with height ratio enhancements. Such an increase in deformation is linked to concurrent deformity in both the coal and rock combinations under the same loading conditions. However, the inherent stiffness of rock is superior to that of coal, resulting in lesser deformation in the rock segment. Hence, within the coal-rock combination samples, an increase in the coal fraction leads to more pronounced deformation.

**Figure 7:**
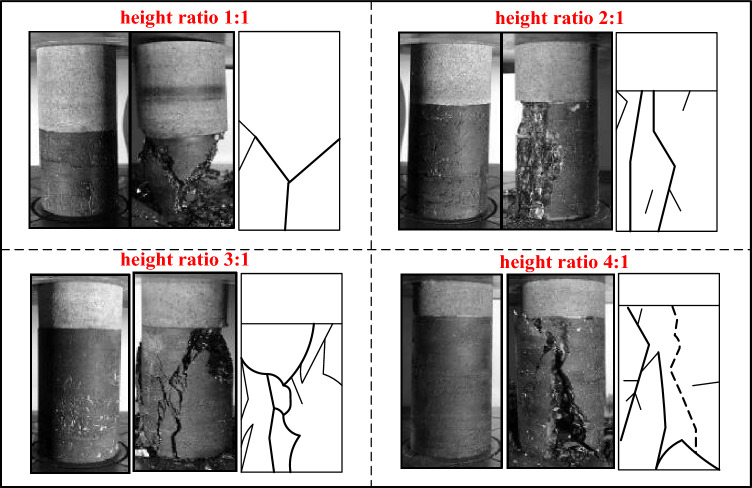
Shape of combinations before and after destruction and the sketch.

Figure 7 shows the shape combinations before and after destruction and their sketches. Macroscopically, the coal–rock combinations with a height ratio of 1:1 eventually fail via single shear plane penetration in the coal body section. Notably, these samples exhibited minimal secondary fracturing, characterized by a "Y"-shaped primary crack, with the bulk of damage localized within the coal region. The coal–rock combinations with height ratios of 2:1, 3:1, and 4:1 all have penetrating primary cracks on their surfaces, with multiple secondary cracks extending near the primary cracks, comprising mostly wing-type cracks, and rougher joint surfaces for tensile failure. Destruction in the whole system was mainly concentrated in the coal body part, and multiple cracks were produced on the surface with spalling of coal pieces. The rock part did not produce prominent cracks. Therefore, the integrity of the coal pillars is critical to the overall structural stability.

**Figure 8:**
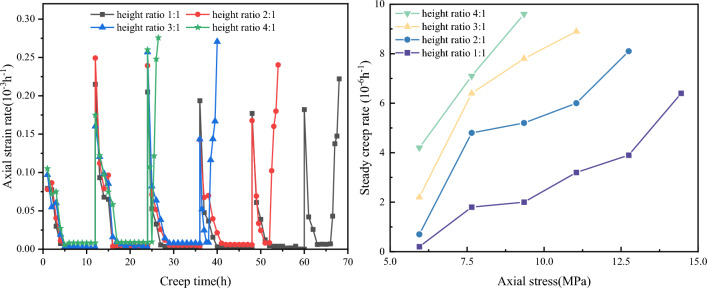
Strain rate curves of coal–rock combinations with different height ratios.

The creep curves of four height ratios were plotted to obtain creep rate curves of coal–rock combinations with different height ratios, and the results are shown in Fig. 8. Noticeably, the left side of the creep rate curve represents the initial creep stage, and the rate of the initial creep stage gradually increases with the height ratio. The specimens then entered the steady creep stage, and the creep rate stabilized to 0.2 × 10^–6^/s–9.6 × 10^–6^/s. With the increase of axial stress and height ratio, the steady creep rate both show a gradual increase. The right side of Fig. 8 shows that the creep rate increased significantly in the last level of the stress loading stage, indicating that the specimen entered the accelerated creep stage. As the height ratio increased, the rate of the accelerated creep stage gradually increased, indicating that the instability failure of the specimen was imminent. The overall curve trend shows that the creep rate curve in the accelerated creep stage is “V”-shaped, and the creep rate curve in the unaccelerated creep stage is “L”-shaped.

To further investigate the creep damage properties of coal-rock combinations , a creep damage model for the coal-rock combinations was established, starting from the creep constitutive model and grounded on the theory of rock mechanics, damage mechanics, and fractional calculus.

## Creep damage model of coal-rock combinations

As indicated by the creep test curves of the composite coal-rock body:

1. The coal–rock combinations undergo immediate elastic deformation upon loading, which requires the inclusion of an elastic element in the model.
2. The creep curve rises slowly after a loading period, corresponding to the initial creep stage, and the viscoelastic characteristics are prominent. Thus, a Kelvin body must be present in the model.
3. The creep curve rises slowly and then tends to even out in the steady creep stage with marked viscous characteristics. Thus, the model contains a viscous body.
4. If the coal–rock combinations are subjected to high stress, the creep curve will exhibit a steady state followed by an accelerated rise, entering the accelerated creep stage with distinct viscoplastic characteristics, indicating imminent creep failure; hence, the model should incorporate a plastic element. The following describes the block elements utilized in the model.

**Figure 9:**
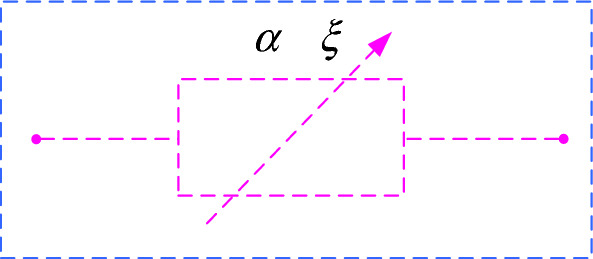
Abel dashpot.

In recent years, the Abel dashpot has been widely used in creep models due to its time-dependent properties. The Abel dashpot is shown in Fig. 9^36^.

The constitutive equation for the Abel dashpot is as follows:

1 $$ \sigma (t) = \xi \frac{{d^{\alpha } \varepsilon (t)}}{{dt^{\alpha } }},\quad (0 \le \alpha \le 1) $$

where $\xi$ is the viscosity coefficient of the Abel dashpot and $\alpha$ is the fractional order.

When $\alpha = 1$ and $\sigma (t) = \xi \cdot \dot{\varepsilon }(t)$, $\dot{\varepsilon }(t)$ is the strain rate, and the Abel dashpot is equivalent to a Newton viscous body, which represents an ideal fluid. When $\alpha = 0$ and $\sigma (t) = \xi \cdot \varepsilon (t)$, the Abel dashpot is equivalent to an elastomer, which represents an ideal solid. When $0 < \alpha < 1$, the Abel dashpot represents an object between an ideal solid and an ideal fluid.

When $\sigma (t) = const$, the creep equation for the Abel dashpot is obtained by performing Riemann–Liouville type fractional calculus^37^ for both sides of Eq. (1) with constant stresses.

The integral of order $\alpha$ of a function $f(x)$ is defined as follows:

2 $$ \frac{{d^{ - \alpha } [f(x)]}}{{dx^{ - \alpha } }} = {}_{{x_{0} }}D_{x}^{ - \alpha } f(x) = \frac{1}{\Gamma (\alpha )}\int_{{x_{0} }}^{x} {(x - t)^{\alpha - 1} } f(t)dt. $$

*x* and *x*_0_ represent the upper and lower limits of integration, respectively. $\Gamma$ is the Gamma function^38^, where $\Gamma (z) = \int_{0}^{\infty } {t^{z - 1} } e^{ - t} dt$.

The Laplace transform formulas for fractional calculus are as follows:

3 $$ \left\{ \begin{gathered} L\left[ {{}_{0}D_{t}^{ - \alpha } f(t),\kappa } \right] = \kappa^{ - \alpha } \overline{f}(\kappa )(\alpha > 0) \hfill \\ L\left[ {{}_{0}D_{t}^{\alpha } f(t),\kappa } \right] = \kappa^{\alpha } \overline{f}(\kappa )(0 \le \alpha \le 1) \hfill \\ \end{gathered} \right., $$

where the Laplace transform of $f(t)$ is denoted as $\overline{f}(\kappa )$^39^.

Referring to Eqs. (2, 3), the integral yields the creep equation for the Abel dashpot as follows:

4 $$ \varepsilon (t) = \frac{\sigma }{\xi }\frac{{t^{\alpha } }}{\Gamma (1 + \alpha )},(0 \le \alpha \le 1) $$

**Figure 10:**
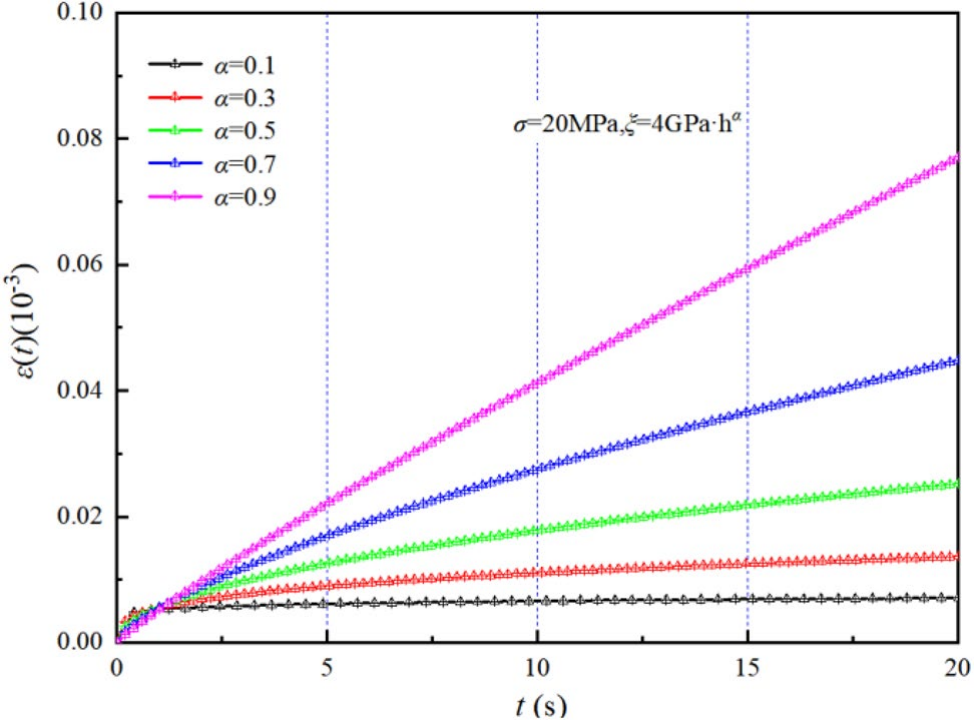
Abel dashpot creep curves with different *α* values.

When $\sigma = 20{\text{MPa,}}\xi = {\text{4GPa}} \cdot {\text{h}}^{\alpha }$, the Origin software custom function is used to plot creep curves for different $\alpha$ values, as shown in Fig. 10. Noticeably, the Abel dashpot can theoretically characterize the steady creep stage. Compared with the Newton viscous body, the Abel dashpot has more nonlinear characteristics.

**Figure 11:**
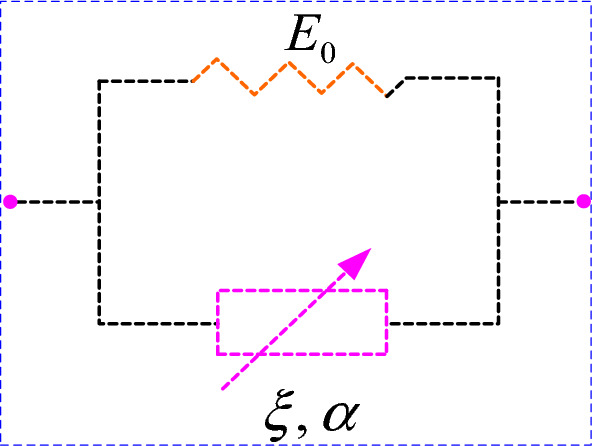
Fractional Kelvin body.

If the Newton viscous body in the conventional Kelvin body is replaced by an Abel dashpot, a fractional Kelvin body is obtained from the Abel viscous pot and the elastomer in parallel, as shown in Fig. 11.

The constitutive equation for the fractional Kelvin body is as follows:

5 $$ \sigma (t) = E_{0} \varepsilon (t) + \xi \frac{{d^{\alpha } \varepsilon (t)}}{{dt^{\alpha } }},\quad (0 \le \alpha \le 1) $$

Similarly, based on Eqs. (2, 3), the creep equation for the fractional Kelvin body can be obtained as follows:

6 $$ \varepsilon (t) = \frac{\sigma }{{E_{0} }}t^{\alpha } E_{\alpha ,\alpha + 1} \left( { - \xi \frac{{t^{\alpha } }}{{E_{0} }}} \right),\quad (0 \le \alpha \le 1) $$

where $E_{i,j} \left( Z \right)$ is the two-parameter Mittag–Leffler function^40^, and the specific expression is

7 $$ E_{i,j} \left( Z \right) = \sum\limits_{n = 0}^{\infty } {\frac{{Z^{n} }}{{\Gamma \left( {ni + j} \right)}}} \quad i > 0,j > 0 $$

**Figure 12:**
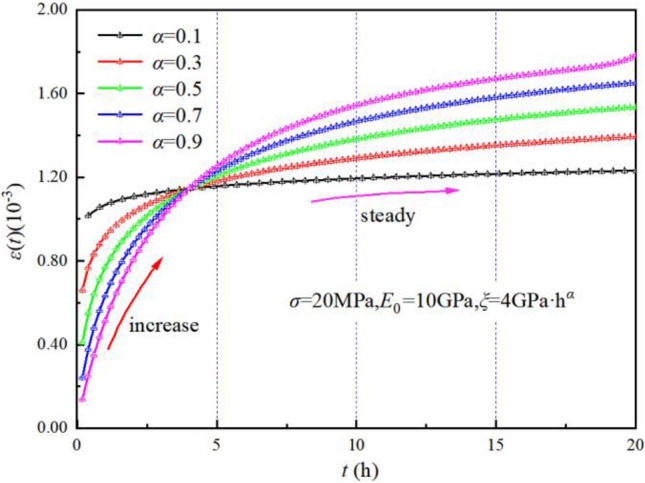
Creep curves of fractional Kelvin with different α values.

When $\sigma = 20{\text{MPa,}}\xi = {\text{4GPa}} \cdot {\text{h}}^{\alpha } {,}E_{0} = {\text{10GPa}}$, the Origin software custom function is used to make creep curves for different $\alpha$ values, as shown in Fig. 12, which reveals that the fractional Kelvin body can theoretically characterize the initial creep stage.

During the deformation of the coal–rock combinations, a steady creep stress threshold *σ*_s1_ and an accelerated creep stress threshold *σ*_s2_ exist. When the stress level exceeds this threshold, distinct steady creep and accelerated creep stages become apparent. When the creep deformation of the specimen changes very little or increases nearly linearly, the steady creep stress threshold has been reached. This experiment was set up with a difference of 1.70 MPa for each level of loading, and directly determining the steady creep stress threshold was not possible. According to Guo^41^, the axial pressure when the specimen enters the steady creep stage minus 1.0 MPa is taken as the steady creep stress threshold *σ*_s1_. According to Yin^7^, a specimen is damaged after the last level of loading is applied for a duration and not under the previous level of loading, indicating that the accelerated creep stress threshold should be between the stress level *σ*_*γ*_ at which damage occurs and the stress level *σ*_*γ*-1_ at the previous level. Moreover, the accelerated creep stress threshold is related to the duration of loading t at the stress level at which damage occurs. The accelerated creep stress threshold *σ*_s2_ is calculated based on the following relation^7^:

8 $$ \sigma_{s2} = \sigma_{\gamma - 1} + \frac{t}{{\text{T}}}\left( {\sigma_{\gamma } - \sigma_{\gamma - 1} } \right). $$

where *t* is the duration for which the specimen is loaded at stress *σ*_*γ*_ and T is the duration of each level of stress loading, taken here as 12 h. The steady and accelerated creep stress thresholds were obtained according to the above method and are listed in Table 1.

**Table 1:** Steady creep stress threshold and accelerated creep stress threshold.

Height ratio	Steady creep stress threshold (MPa)	Accelerated creep stress threshold (MPa)
1:1	10.05	14.025
2:1	8.35	11.90
3:1	6.65	9.917
4:1	4.95	8.004

The test data reveal that when the axial stress reaches the accelerated creep stress threshold *σ*_s2_, the accelerated creep stage does not occur immediately, but the immediate deformation, initial creep, and steady creep stage occur first, and only after a period will the accelerated creep stage commence, indicating that creep instability has hysteresis characteristics. This study defines the moment when the accelerated creep stage is reached after a period *t*_F_, which can be experimentally determined. Therefore, the constraint for the specimen entering the accelerated creep stage is *σ* ≥ *σ*_s2_ and *t* ≥ *t*_F_.

**Figure 13:**
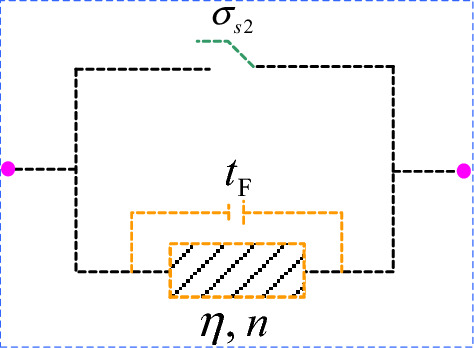
Modified nonlinear viscoplastic body.

The model is improved based on the nonlinear viscoplastic body (NVPB), as shown in Fig. 13.

Its creep equation is as follows

9 $$ \varepsilon \left( t \right) = \left\{ \begin{gathered} \quad \quad \quad 0\quad \quad \quad \;{\kern 1pt} {\kern 1pt} {\kern 1pt} {\kern 1pt} t < t_{{\text{F}}} \quad or\quad \sigma < \sigma_{s2} \hfill \\ \frac{{\sigma - \sigma_{s2} }}{\eta }\left( {t - t_{{\text{F}}} } \right)^{n} \quad t \ge t_{{\text{F}}} \quad {\text{and}}\quad \sigma \ge \sigma_{s2} \hfill \\ \end{gathered} \right.. $$

**Figure 14:**
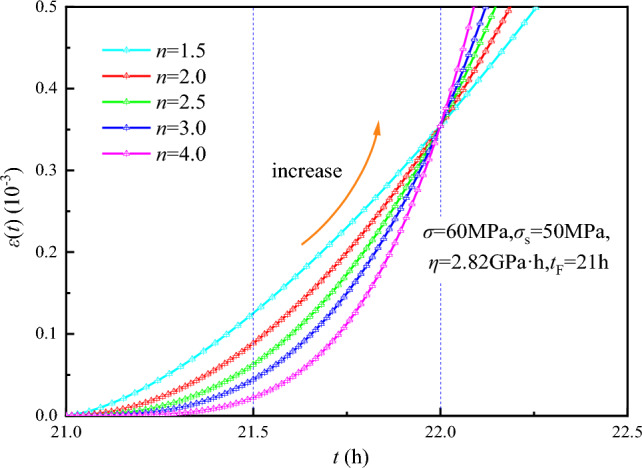
Creep curves of modified nonlinear viscoplastic body with different *n* value.

The creep curves for different values of *n* when $$\left\{ \begin{gathered} \sigma = 60{\text{MPa,}}\sigma_{s2} = 50{\text{MPa,}} \hfill \\ \eta = {2}.82{\text{GPa}} \cdot {\text{h,}}t_{{\text{F}}} = 21{\text{h}} \hfill \\ \end{gathered} \right.$$ are plotted in Fig. 14. As shown in Fig. 14, the modified NVPB body can theoretically characterize the accelerated creep stage.

According to the test data in Fig. 6, the larger the height ratio, the earlier creep instability occurs in the coal–rock combinations. Differences also occur in the deformation of coal–rock combinations at different stress levels and times. This indicates that the creep damage of coal–rock combinations is associated with the height ratio, stress, and time. Therefore, the creep damage of the coal–rock combinations is defined as *D*(*σ*,*h*,*t*). The damage produced during the stress loading transient is independent of time, whereas the damage produced afterward is time-dependent, so the following creep damage expression is established:

10 $$ D\left( {\sigma ,h,t} \right) = 1 - \left[ {1 - D\left( {\sigma ,h} \right)} \right]{\text{e}}^{ - \lambda t} . $$

where *D*(*σ*,*h*) is the instantaneous damage, a function related to stress and height ratio; *h* is the dimensionless height ratio, which is the ratio of coal volume to rock volume; and *λ* is the damage parameter. When *t* = 0, *D*(*σ*,*h*,*t*) = *D*(*σ*,*h*), and when $t \to \infty$, $D\left( {\sigma ,h,t} \right) \to 1$.

This paper defines the damage variable based on the change in elastic modulus. The initial elastic modulus is set as *E*_0_, and the *E*_0_ values under different stresses and height ratios are calculated from the instantaneous deformation obtained from the experimental data, as listed in Table 2.

**Table 2:** *E*_0_ values of different stress and height ratios.

Axial stress (MPa)	Height ratio			
	1:1	2:1	3:1	4:1
5.95	3.71	2.57	2.18	1.88
7.65	3.79	2.75	2.44	2.08
9.35	3.86	2.79	2.49	2.14
11.05	3.98	2.99	2.66	–
12.75	4.14	3.10	–	–
14.45	4.26	–	–	–

The following equation is obtained from $E = E_{0} \times \left[ {1 - D\left( {\sigma ,h} \right)} \right]$:

11 $$ D\left( {\sigma ,h} \right) = 1 - \frac{E}{{E_{0} }} $$

The expression of the function of *D*(*σ*,*h*) obtained by substituting the data for the nonlinear surface fit is as follows:

12 $$ D\left( {\sigma ,h} \right) = \frac{0.34843h\sigma }{{\sqrt {\sigma^{2} + 287.13h} }} - 0.08041,R^{2} = 0.912 $$

The expression for the damage variable *D*(*σ*,*h*,*t*) is obtained by substituting Eq. (12) into Eq. (10).

**Figure 15:**
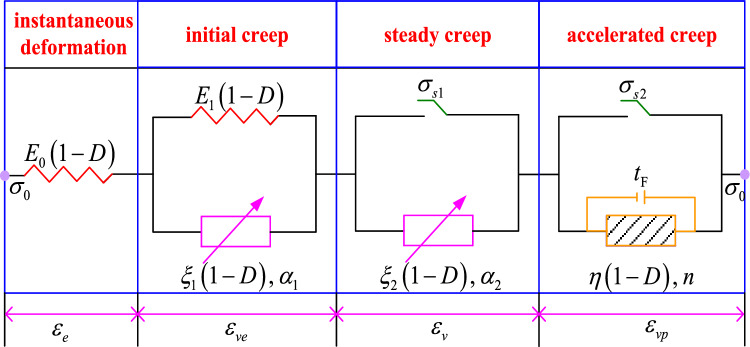
Fractional creep damage model.

A creep damage model for coal–rock combinations is established from the above, as shown in Fig. 15.

The following assumptions are made in deriving the creep equation:

1. The composite coal body is an isotropic material with consistent damage in all directions.
2. The damage duration was consistent with the corresponding creep time.

The creep equations of the creep damage model of the coal–rock combinations are derived according to the principle of series–parallel connection of elements and the superposition principle.

1. $\sigma_{0} < \sigma_{s1}$ corresponds to the initial creep stage. The model is equivalent to an elastomer connected in series with a fractional Kelvin body. The constitutive equation of the creep model is given by
  13 $$ \left\{ \begin{gathered} \sigma_{0} = E_{0} \left( {1 - D} \right)\varepsilon_{e} \hfill \\ \sigma_{0} = E_{1} \left( {1 - D} \right)\varepsilon_{ve} + \xi_{1} \left( {1 - D} \right)\frac{{d^{{\alpha_{1} }} \varepsilon_{ve} }}{{dt^{{\alpha_{1} }} }} \hfill \\ \varepsilon (t) = \varepsilon_{e} + \varepsilon_{ve} \hfill \\ \end{gathered} \right.. $$
2. $\sigma_{s1} \le \sigma_{0} < \sigma_{s2}$ corresponds to the steady creep stage. The constitutive equation of the creep model is given by
  14 $$ \left\{ \begin{gathered} \sigma_{0} = E_{0} \left( {1 - D} \right)\varepsilon_{e} \hfill \\ \sigma_{0} = E_{1} \left( {1 - D} \right)\varepsilon_{ve} + \xi_{1} \left( {1 - D} \right)\frac{{d^{{\alpha_{1} }} \varepsilon_{ve} }}{{dt^{{\alpha_{1} }} }} \hfill \\ \sigma_{0} - \sigma_{s1} = \xi_{2} \left( {1 - D} \right)\frac{{d^{{\alpha_{2} }} \varepsilon_{v} }}{{dt^{{\alpha_{2} }} }} \hfill \\ \varepsilon (t) = \varepsilon_{e} + \varepsilon_{ve} + \varepsilon_{v} \hfill \\ \end{gathered} \right.. $$
3. $\sigma_{0} \ge \sigma_{s2} \;{\text{and}}\;t \ge t_{F}$ correspond to the accelerated creep stage. The constitutive equation of the creep model is given by
  15 $$ \left\{ \begin{gathered} \sigma_{0} = E_{0} \left( {1 - D} \right)\varepsilon_{e} \hfill \\ \sigma_{0} = E_{1} \left( {1 - D} \right)\varepsilon_{ve} + \xi_{1} \left( {1 - D} \right)\frac{{d^{{\alpha_{1} }} \varepsilon_{ve} }}{{dt^{{\alpha_{1} }} }} \hfill \\ \sigma_{0} - \sigma_{s1} = \xi_{2} \left( {1 - D} \right)\frac{{d^{{\alpha_{2} }} \varepsilon_{v} }}{{dt^{{\alpha_{2} }} }} \hfill \\ \sigma_{0} - \sigma_{s2} = \frac{{\eta \left( {1 - D} \right)\varepsilon_{vp} }}{{\left( {t - t_{{\text{F}}} } \right)^{n} }} \hfill \\ \varepsilon (t) = \varepsilon_{e} + \varepsilon_{ve} + \varepsilon_{v} + \varepsilon_{vp} \hfill \\ \end{gathered} \right.. $$

In combination with Eqs. (13, 14, 15), the one-dimensional creep damage equation of the coal–rock combinations is obtained as

16 $$ \varepsilon \left( t \right) = \left\{ \begin{gathered} \frac{{\sigma_{0} }}{{E_{0} \left( {1 - D} \right)}} + \frac{{\sigma_{0} t^{{\alpha_{1} }} }}{{E_{1} \left( {1 - D} \right)}}E_{{\alpha_{1} ,\alpha_{1} + 1}} \left[ { - \xi_{1} \left( {1 - D} \right)\frac{{t^{{\alpha_{1} }} }}{{E_{1} }}} \right]\quad \sigma_{0} < \sigma_{s1} \hfill \\ \frac{{\sigma_{0} }}{{E_{0} \left( {1 - D} \right)}} + \frac{{\sigma_{0} t^{{\alpha_{1} }} }}{{E_{1} \left( {1 - D} \right)}}E_{{\alpha_{1} ,\alpha_{1} + 1}} \left[ { - \xi_{1} \left( {1 - D} \right)\frac{{t^{{\alpha_{1} }} }}{{E_{1} }}} \right] + \frac{{\left( {\sigma_{0} - \sigma_{s1} } \right)\left( {t - t_{{\text{d}}} } \right)^{{\alpha_{2} }} }}{{\xi_{2} \left( {1 - D} \right)\Gamma \left( {1 + \alpha_{2} } \right)}}\quad \sigma_{s1} \le \sigma_{0} < \sigma_{s2} \hfill \\ \frac{{\sigma_{0} }}{{E_{0} \left( {1 - D} \right)}} + \frac{{\sigma_{0} t^{{\alpha_{1} }} }}{{E_{1} \left( {1 - D} \right)}}E_{{\alpha_{1} ,\alpha_{1} + 1}} \left[ { - \xi_{1} \left( {1 - D} \right)\frac{{t^{{\alpha_{1} }} }}{{E_{1} }}} \right] + \frac{{\left( {\sigma_{0} - \sigma_{s1} } \right)\left( {t - t_{{\text{d}}} } \right)^{{\alpha_{2} }} }}{{\xi_{2} \left( {1 - D} \right)\Gamma \left( {1 + \alpha_{2} } \right)}} + \frac{{\left( {\sigma_{0} - \sigma_{s2} } \right)\left( {t - t_{{\text{F}}} } \right)^{n} }}{{\eta \left( {1 - D} \right)}} \hfill \\ \quad \quad \quad \quad \quad \quad \quad \quad \quad \quad \quad \quad \quad \quad \quad \quad \quad \quad \quad \quad \quad \quad \sigma_{0} \ge \sigma_{s2} \;and\;t \ge t_{{\text{F}}} \hfill \\ \end{gathered} \right.. $$

where, *σ*_0_ is the axial stress, *E*_0_ is the initial elastic modulus, *E*_1_ is the viscoelastic modulus, *α*_1_ is the initial creep fractional order, *ξ*_1_ is the initial creep fractional viscosity coefficient, *α*_2_ is the steady creep fractional order, *ξ*_2_ is the steady creep fractional viscosity coefficient, *σ*_s1_ is the steady creep stress threshold, *σ*_s2_ is the accelerated creep stress threshold, *t*_d_ is the steady creep initiation time, *t*_F_ is the accelerated creep initiation time, *η* is the accelerated creep viscosity coefficient, and *n* is the accelerated creep index.

## Parameter identification and model validation

The parameters modeled in the article are *E*_0_, *E*_1_, *α*_1_, *ξ*_1_, *α*_2_, *ξ*_2_, *η*, *n*, and *λ*. The determination of these model parameters is also crucial. Parameter *E*_0_ is determined from transient deformation test data. Parameters *E*_1_, *α*_1_, *ξ*_1_, *α*_2_, *ξ*_2_, *η*, *n*, and *λ* are determined by fitting the test data. Because of its complexity, the creep equation could not be directly fitted using the original function in Origin software. The specific procedure involves selecting [Nonlinear Curve Fitting] in the [Fitting] toolbar and then writing [LabTalk Expression] to complete the user-defined function fitting. To avoid nonconvergence or a local optimum due to too many parameters and improper selection of initial values, a modified nonlinear least squares method based on pattern search was used to invert the creep parameters *E*_1_, *α*_1_, *ξ*_1_, *α*_2_, *ξ*_2_, *η*, *n*, and *λ*^42^. To demonstrate the effectiveness of the model established in the paper, the aforementioned parameter inversion method was applied for parameter identification on creep test data of coal-rock combinations with four different height ratios. The model parameters obtained are shown in Table 3.

**Table 3:** Results table of creep parameter inversion.

Axial stress (MPa)	*E*_0_ (GPa)	*E*_1_ (GPa)	*α*_1_	*ξ*_1_ (GPa·s^*α*1^)	*α*_2_	*ξ*_2_ (GPa·s^*α*2^)	*η *(GPa·h)	*n*	*λ *(10^–4^)	R^2^
Model parameters for a height ratio of 1:1										
5.95	3.71	0.38	0.24	2.24	–	–	–	–	0.132	0.954
7.65	3.79	0.46	0.27	2.57	–	–	–	–	0.147	0.962
9.35	3.86	0.53	0.31	3.02	–	–	–	–	0.162	0.959
11.05	3.98	0.72	0.32	3.13	0.86	5.52	–	–	0.177	0.973
12.75	4.14	0.96	0.35	3.28	0.88	5.71	–	–	0.192	0.976
14.45	4.26	1.01	0.41	3.76	0.90	5.93	9.86	4.42	0.207	0.968
Model parameters for a height ratio of 2:1										
5.95	2.57	0.64	0.25	2.51	–	–	–	–	0.133	0.956
7.65	2.75	0.77	0.27	2.83	–	–	–	–	0.148	0.974
9.35	2.79	0.92	0.32	3.17	0.73	4.96	–	–	0.163	0.968
11.05	2.99	0.99	0.36	3.38	0.88	5.43	–	–	0.178	0.966
12.75	3.10	1.13	0.43	3.69	0.92	5.87	8.93	5.04	0.193	0.972
Model parameters for a height ratio of 3:1										
5.95	2.18	0.73	0.27	2.58	–	–	–	–	0.134	0.988
7.65	2.44	0.86	0.28	2.96	0.79	4.88	–	–	0.149	0.984
9.35	2.49	0.97	0.37	3.24	0.89	5.24	–	–	0.164	0.965
11.05	2.66	1.20	0.41	3.56	0.92	5.46	8.28	5.26	0.179	0.979
Model parameters for a height ratio of 4:1										
5.95	1.88	0.77	0.34	2.86	0.81	4.52	–	–	0.135	0.972
7.65	2.08	0.91	0.48	3.24	0.84	5.13	–	–	0.150	0.981
9.35	2.14	1.34	0.51	4.67	0.94	5.37	7.63	5.41	0.165	0.968

**Figure 16:**
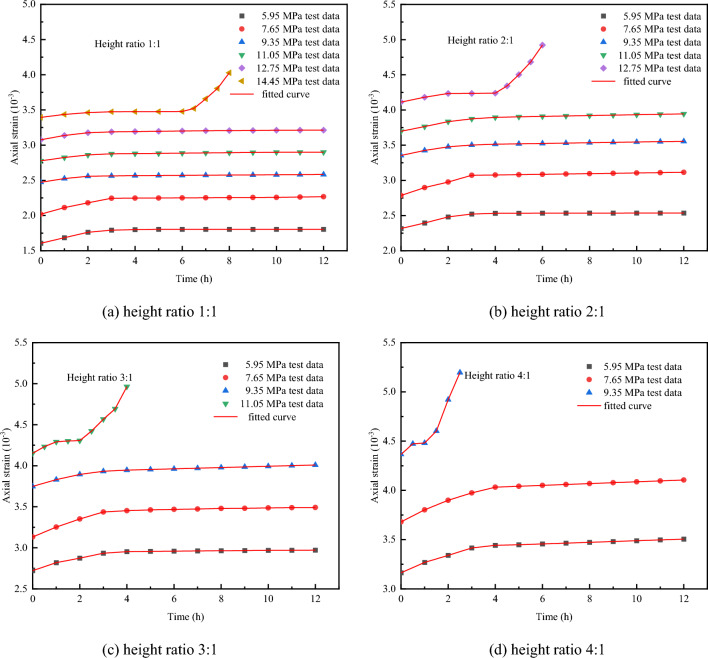
Comparison between the creep test results and the theoretical curve of coal–rock combinations.

The comparison between the creep test results and the theoretical curve of coal–rock combinations is shown in Fig. 16.

**Figure 17:**
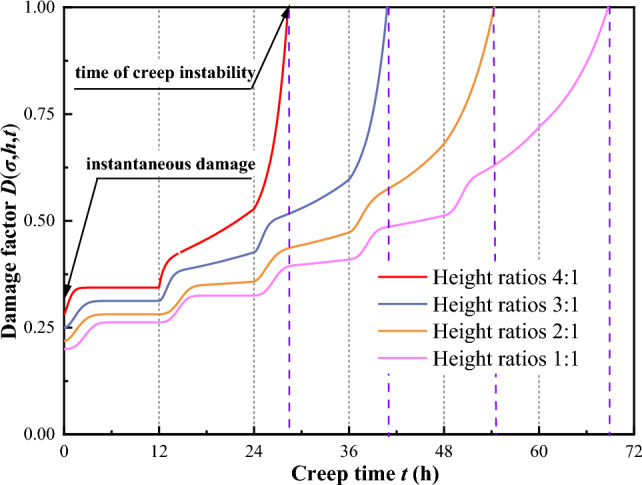
Damage curves of coal–rock combinations with different height ratios.

As can be seen from Fig. 16, the model curves are well-fitted, with coefficients of determination all above 0.95. Additionally, the established fractional creep damage model is demonstrated to be capable of describing not only the instantaneous deformation and the steady creep characteristics of coal–rock combinations with varying height ratios but also the initial creep behavior and the accelerating creep behavior during the nonlinear stage, thereby substantiating the model's effectiveness. The creep parameters obtained from the inversion were substituted into Eq. (10) to produce the damage curves for different height ratios, as shown in Fig. 17.

Figure 17 reveals that the curve intercept is the instantaneous damage. With time, the damage accumulates and tends to 1, and the specimen is destroyed. Moreover, as the height ratio increased, the damage accumulated faster. The time when the damage reaches the maximum in the curve is similar to the time of creep instability, which illustrates the reasonableness of the damage expression established.

**Figure 18:**
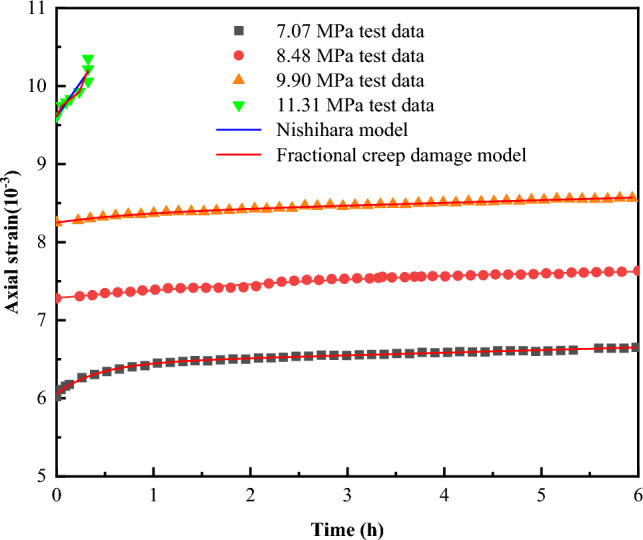
Fitted curves of coal–rock combination in reference^7^.

To verify the generalizability of the model, creep data of coal–rock combinations from the literature^7^ were fitted, as shown in Fig. 18.

As can be seen from Fig. 18, the fractional creep damage model established in this paper is capable of describing the entire creep process of the coal–rock combination specimens from reference^7^ quite effectively. Moreover, during the accelerated creep phase, the fractional-order creep damage model exhibits a higher degree of fit compared to the Nishihara model^43^. In summary, the fractional creep damage model developed in the paper is reasonable and valid.

## Relationship between model parameters and test conditions

To explore the relationship between model parameters and the height ratio and axial stress, a multivariate nonlinear regression analysis was carried out using the data presented in Table 3. This analysis established the correlation of model parameters (*E*_0_, *E*_1_, *α*_1_, *ξ*_1_, *α*_2_, *ξ*_2_, *η*, *n*, *λ*) with height ratio and axial stress, as shown in Eqs. (17–25).

17 $$ E_{0} = 1138.49 + 0.07052h - 1135.30\sigma^{0.00111} ,R^{2} = 0.986 $$

18 $$ E_{1} = - 87.71 + 0.08447h + 87.52\sigma^{0.00439} ,R^{2} = 0.923 $$

19 $$ \alpha_{1} = 0.07536 + 0.02411h + 7.47 \times 10^{ - 4} \sigma^{3.95805} ,R^{2} = 0.912 $$

20 $$ \xi_{1} = 1.05348 + 0.19122h + 0.02645\sigma^{2.64484} ,R^{2} = 0.892 $$

21 $$ \alpha_{2} = 0.39362 + 0.03611h + 0.01958\sigma^{1.66007} ,R^{2} = 0.879 $$

22 $$ \xi_{2} = 3.11347 + 0.20235h + 0.01344\sigma^{2.32316} ,R^{2} = 0.942 $$

23 $$ \eta = - 1.62826 + 0.74928h + 0.53997\sigma ,R^{2} = 0.940 $$

24 $$ n = 1.8065 + 0.14991h + 0.57384\sigma ,R^{2} = 0.914 $$

25 $$ \lambda = 0.07835 + 0.00103h + 0.008775\sigma ,R^{2} = 0.988 $$

**Figure 19:**
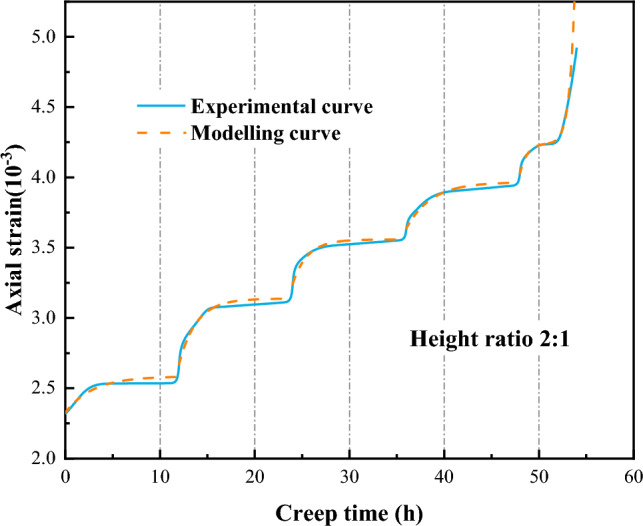
Comparison of modelling curve and experimental curve.

The model parameters under different height ratios and axial stress conditions can be calculated by using the Eqs. (17–25). The calculated model parameters are substituted into Eq. (16) and combined with the stress threshold calculation equation to obtain the creep curve of the coal-rock combinations at different test conditions. This method is now applied to predict the creep curve for a height ratio of 2:1, and the comparison between the modeling curve and the experimental curve is illustrated in Fig. 19. As can be seen from Fig. 19, the general trend of the modeling curve is similar to that of the experimental curve. The reason for the error in the numerical magnitude is that the fitted relational equation of the parameters itself is in error, so the obtained curve will also have some error, but the overall prediction effect is good.

## Conclusion

To deepen the understanding of the creep characteristics of the coal–rock combinations, uniaxial creep tests were carried out on coal–rock combinations with varying height ratios. A creep damage model for the coal–rock combinations was developed based on the principles of rock mechanics, damage mechanics, and fractional calculus, resulting in the following conclusions:

1. The creep processes for coal–rock combinations with different height ratios are similar. Under the same axial stress condition, with the increase in height ratio, the total deformation of coal–rock combinations increases gradually, and the ultimate load level that can be withstood decreases, the total creep time decreases, the instantaneous deformation increases, the increase in instantaneous deformation decreases, the creep deformation increases, and the creep rate increases. For the same height ratio, with the increase in axial stress, the total deformation of the coal–rock combinations also gradually increases, and the creep deformation tends to increase, decrease, and then increase.
2. The failure of the coal–rock combinations predominantly occurs in the coal portion, with a height ratio of 1:1 coal–rock combinations exhibiting shear failure, while those with height ratios of 2:1, 3:1, and 4:1 undergo tensile failure. The creep rate curves during the accelerated creep stage are "V"-shaped, whereas the creep rate curves without an accelerated creep stage are "L"-shaped.
3. The elastomer, fractional Kelvin body, plastic body, Abel dashpot, and modified NVPB body are combined, and the damage variable *D*(*σ*,*h*,*t*) related to stress, height ratio, and time is introduced. The creep damage model of the coal–rock combinations is established, the one-dimensional creep equation is derived, and the parameter inversion method is established.
4. The parameter identification results demonstrate that the creep model established within this paper effectively captures the full creep behavior of coal–rock combinations. The conformity between the modeling curves and the experimental data is notably precise with minimal error. Furthermore, the fit of the model to the creep data of composite coal-rock body samples presented in literature^4^ is proficient, indicating the model’s adaptability. Compared to the Nishihara model, the one developed in this study shows greater fitting accuracy, thereby confirming its viability. Additionally, the model effectively forecasts the creep curve for a specimen with a height to width ratio of 2:1 under an axial stress of 12.80 MPa, with the predictive outcome being satisfactory.

### Ethics approval

The entire content of the article are written in accordance with ethical standards.

## Data Availability

The data that support the findings of this study are available from the corresponding author upon reasonable request.
